# Acrokeratosis paraneoplastica (Bazex syndrome): a systematic review and quantitative analysis of 106 cases

**DOI:** 10.1093/oncolo/oyag376

**Published:** 2026-09-24

**Authors:** Mustafa Sefa Isin, Mehmet Berke Isler, Amor Khachemoune

**Affiliations:** International School of Medicine, Istanbul Medipol University, Istanbul, 34815, Turkey; International School of Medicine, Istanbul Medipol University, Istanbul, 34815, Turkey; Premier Dermatology, Ashburn, VA 20147, USA; Department of Dermatology, Istanbul Medipol University, International School of Medicine, Istanbul, 34214, Turkey

**Keywords:** Bazex syndrome, Acrokeratosis paraneoplastica, Paraneoplastic dermatosis, Squamous cell carcinoma, Psoriasiform lesions

## Abstract

**Background:**

Acrokeratosis paraneoplastica (Bazex syndrome) is an obligate paraneoplastic dermatosis characterized by symmetrical acral psoriasiform lesions and nail dystrophy.

**Methods:**

We conducted a PRISMA-compliant systematic review of 99 publications (89 case reports, 10 case series) describing 106 patients to re-examine the syndrome’s epidemiology, oncologic associations, clinical features, treatment response, and prognosis.

**Results:**

The mean age at onset was 63.5 years (median: 62.5 years; interquartile range [IQR]: 57-70 years); men comprised 72.6% (*n* = 77; 95% CI, 63.5%-80.2%), yielding a male-to-female ratio of 2.7:1. Squamous cell carcinoma accounted for 51.9% (55/106; 95% CI, 42.5%-61.2%) of associated malignancies, followed by adenocarcinomas (18.9%; 95% CI, 12.6%-27.4%) and hematologic neoplasms (8.5%; 95% CI, 4.5%-15.4%). Cutaneous lesions preceded the cancer diagnosis in 80.6% (83/103) of patients with reported chronology, with a median lead-time of 5 months (IQR 2-10 months). Nail involvement was documented in 73.6% (78/106). Following oncologic therapy, complete cutaneous clearance occurred in 36.8% (95% CI, 28.2%-46.3%) and partial improvement in 30.2% (95% CI, 22.3%-39.5%) of cases. In univariable analyses, distant metastasis (*P* = .021) and an aerodigestive primary site (*P* = .026) were nominally associated with reported mortality, although neither remained significant after multiple-comparison correction.

**Conclusion:**

Bazex syndrome should be suspected in patients presenting with treatment-resistant acral psoriasiform lesions and nail dystrophy, prompting rigorous malignancy screening.

Implications for practice This review broadens the published profile of Bazex syndrome beyond its traditional link to men with aerodigestive squamous cell carcinomas and highlights meaningful associations with gastrointestinal and hematologic malignancies across a wider demographic spectrum; however, this may partly result from publication bias toward atypical presentations. Because refractory acral psoriasiform lesions typically precede tumor detection by several months, early clinical recognition is essential. Clinicians should maintain a high index of suspicion for an underlying malignancy in any patient presenting with these refractory cutaneous lesions. Recurrence of the cutaneous lesions may also provide an early clinical clue to tumor relapse, allowing timely oncologic reassessment.

## Introduction

Acrokeratosis paraneoplastica (Bazex syndrome) is a rare obligate paraneoplastic dermatosis characterized by acral psoriasiform lesions.^1^ First noted as a distinct clinical entity by Gougerot and Rupp^2^ in 1922 and subsequently delineated as a paraneoplastic marker by Bazex et al.^3^ in 1965, the syndrome has since been refined through larger case series and analytical reviews.^4–6^ Cutaneous lesions initially appear as symmetrical erythematous and violaceous plaques on the nose, ear helices, hands, and feet, typically accompanied by severe nail dystrophy.^5–7^ The condition progresses centripetally in clinical stages that closely mirror underlying tumor progression, eventually spreading to the trunk if the malignancy goes untreated.^8–10^

The syndrome is classically associated with squamous cell carcinomas (SCCs) of the upper aerodigestive tract and lung in men older than 40 years.^4–6^ However, the reported oncologic spectrum has expanded to include various non-squamous solid tumors and hematologic malignancies.^11–19^

The pathogenesis remains unclear, likely involving immune dysregulation, epidermal growth factor receptor (EGFR)-pathway activation,^20^ and paraneoplastic tumor–host interactions.^21^

Diagnosis is challenging because the syndrome clinically resembles psoriasis or eczema, and histopathology is nonspecific.^5^^,^^22^ Crucially, cutaneous lesions precede the cancer diagnosis in 60%-70% of patients,^4^^,^^6^ providing a vital window for occult malignancy detection. Cutaneous lesions often improve following successful oncological treatment.^23^

Currently, most primary data are confined to scattered case reports without a comprehensive up-to-date quantitative synthesis. We therefore analyzed 99 publications encompassing 106 individual cases to synthesize the published literature and evaluate the reported spectrum of its demographics, oncological associations, dermatological features, treatment, and prognosis.

## Materials and methods

### Search strategy and selection criteria

We conducted this systematic review in accordance with the Preferred Reporting Items for Systematic Reviews and Meta-Analyses (PRISMA) guidelines.^24^ We searched PubMed (MEDLINE), Scopus, Web of Science, and Wiley Online Library from database inception to November 2025 using the exact search terms: “Bazex syndrome” OR “Acrokeratosis paraneoplastica” OR “Paraneoplastic acrokeratosis.” Duplicate records were identified and removed using Zotero version 7 reference management software, followed by manual verification. We included English-language case reports and case series providing original clinical data. During the initial screening, records were excluded if they were irrelevant to Bazex syndrome or represented an ineligible article type (e.g. animal studies, abstracts lacking core data) (*n* = 293). At the retrieval stage, reports that could not be accessed were excluded (*n* = 27). At the full-text review stage, 5 articles were excluded as they were not available in English. Two reviewers independently screened records and assessed full texts, resolving disagreements by consensus or a 3rd reviewer.

### Data extraction and quality assessment

Two independent reviewers extracted case-level data across 4 domains: demographics, oncology (malignancy type, primary site, stage), dermatology (lesion localization, nail changes, chronological relationship), and outcomes (treatment response, reported mortality). To mitigate the risk of duplicate case reporting, a recognized challenge in rare disease literature spanning several decades, we rigorously cross-referenced patient demographics, oncological characteristics, author affiliations, and geographic locations. Suspected duplicate reports of the same patient were excluded.

Methodological quality was appraised independently by 2 reviewers using the Joanna Briggs Institute (JBI) Critical Appraisal Checklist for Case Reports.^25^ All 99 included publications met at least 5 of the 8 JBI criteria, a threshold chosen to ensure sufficient reporting quality while accommodating short-format reports of patients with rapidly fatal disease courses. Detailed item-specific JBI quality assessment scores for each included case are presented in Table S2. Comprehensive case-level data for all 106 cases are provided in Table S1.

### Statistical analysis

Continuous variables are reported as medians with interquartile ranges (IQRs) alongside means with standard deviations and ranges. Categorical variables were summarized as frequencies and percentages, with 95% confidence intervals (CIs) calculated for key proportions using the Wilson score interval. Closely related histological labels were harmonized into broader categories. Univariable mortality comparisons utilized Fisher’s exact test for categorical variables (metastasis, aerodigestive site, SCC histology, sex, cutaneous lesion chronology) and the Mann–Whitney U test for age. Bonferroni and Benjamini–Hochberg adjusted *P*-values were calculated as sensitivity analyses for multiple comparisons. A 2-sided *P*-value <.05 indicated nominal statistical significance for unadjusted tests. Analyses were performed using Python 3.12 (SciPy 1.12).

## Results

The database search yielded 725 records. After duplicate removal, title/abstract screening, and full-text assessment, 99 publications (89 case reports, 10 case series) comprising 106 cases were included (Figure 1).^4^^,^^9–20^^,^^22^^,^^23^^,^^26–108^

**Figure 1.**
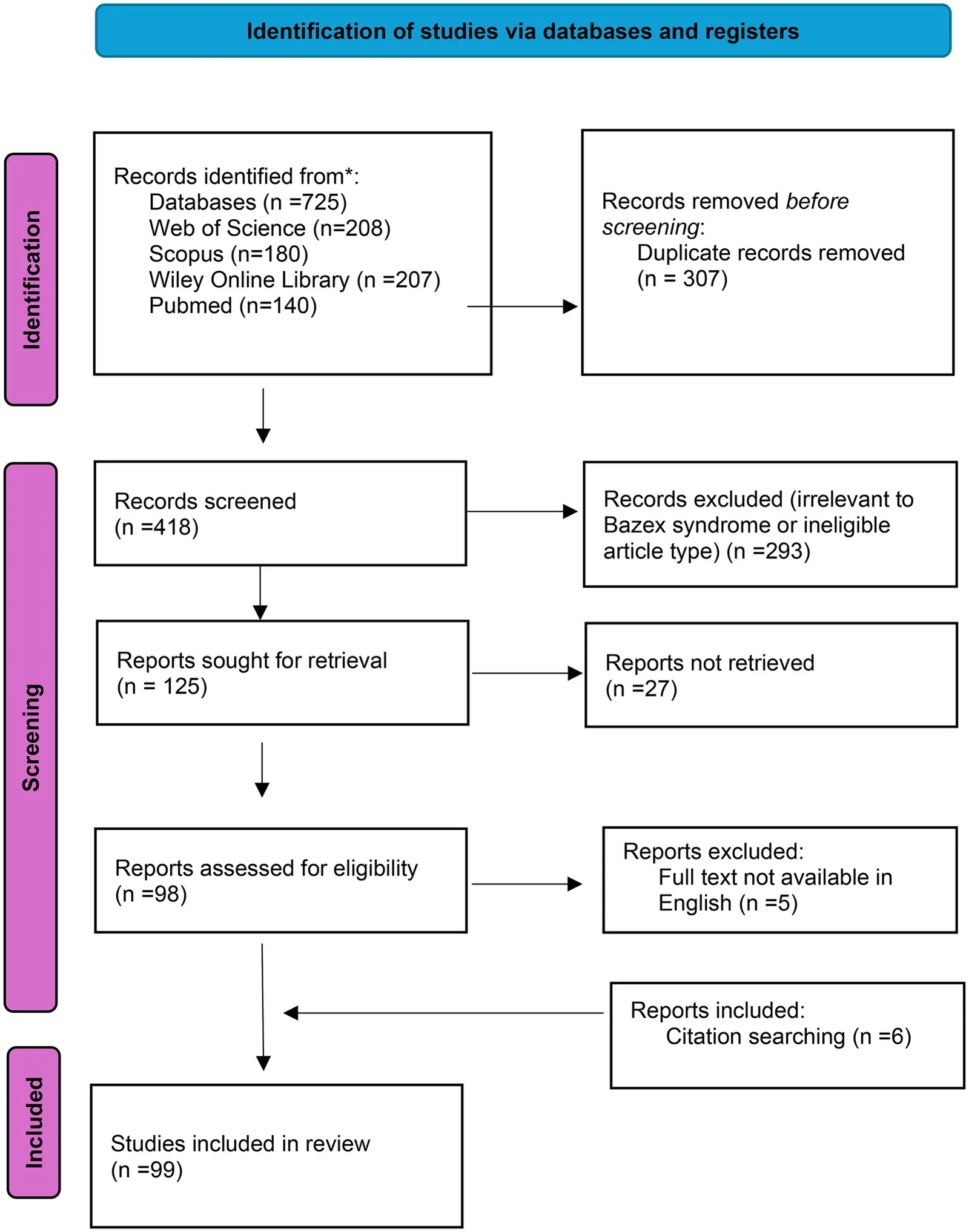
PRISMA flow diagram of study selection.

### Patient demographics and epidemiology

The mean age at onset was 63.5 years (median: 62.5 years; IQR: 57-70 years). Age at onset ranged from 40 to 84 years, with the oldest patients typically presenting with advanced lung or laryngeal cancers.^4^^,^^26–28^ Men comprised 72.6% (*n* = 77; 95% CI, 63.5%-80.2%), and women 27.4% (*n* = 29; 95% CI, 19.8%-36.5%) of the dataset yielding a male-to-PD ratio of 2.7:1. While men predominantly presented with aerodigestive SCC, women exhibited a broader spectrum of non-aerodigestive primary tumors, including gastrointestinal (GI), gynecologic, and breast malignancies.^14^^,^^15^^,^^29–34^

The syndrome demonstrated a global distribution across Caucasian, Asian, African American, Hispanic, and Brazilian populations. Notably, in African American and Black patients, the characteristic violaceous erythema frequently presented as pronounced hyperpigmentation,^11^^,^^17^ though classical palmoplantar keratoderma and nail dystrophy remained clinically indistinguishable across all groups (Table S1).

Tobacco use was the predominant risk factor, with cumulative smoking burdens ranging from 1.25 to 180 pack-years.^28^ Heavy smoking was often accompanied by severe alcohol abuse, particularly in head-and-neck or esophageal cancers.^35–40^ Occupational exposures were rarely reported; only one patient cited prior industrial gasoline contact.^41^ Consequently, common comorbidities reflected this risk profile: alcohol-related liver disease,^42^^,^^43^ cardiovascular conditions (hypertension, prior myocardial infarction, non-ischemic cardiomyopathy),^44^^,^^45^ and pulmonary diseases (chronic obstructive pulmonary disease, idiopathic pulmonary fibrosis).^9^^,^^38^^,^^46^ Less common comorbidities included type 2 diabetes,^13^^,^^47^ Porphyria Cutanea Tarda,^15^ and remitting seronegative symmetrical synovitis with pitting edema (RS3PE) syndrome.^47^ Additionally, a few patients had a history of an unrelated malignancy.^4^^,^^48^

### Oncological characteristics

#### Distribution of primary malignancies and histological subtypes

Pooled data from 106 cases demonstrated SCC as the most frequently reported histology (51.9%, *n* = 55; 95% CI, 42.5%-61.2%), followed by adenocarcinoma/glandular carcinoma (18.9%, *n* = 20; 95% CI, 12.6%-27.4%) (Table 1). The remaining histologies 29.2% (*n* = 31) comprised hematologic malignancies (8.5%, *n* = 9), small cell/neuroendocrine carcinomas (1.9%, *n* = 2), hepatocellular carcinoma (1.9%, *n* = 2), sarcomas (1.9%, *n* = 2), and other or unclassifiable histologies (15.1%, *n* = 16) (Table 1).

**Table 1.** Distribution of histological subtypes among 106 patients with Bazex syndrome.

Histological category	*n*	%
**Squamous cell carcinoma (all variants)**	55	51.9
**Adenocarcinoma/glandular carcinoma**	20	18.9
**Hematologic malignancies/clonal disorders^a^**	9	8.5
**Small cell/neuroendocrine carcinoma**	2	1.9
**Hepatocellular carcinoma**	2	1.9
**Sarcoma (angiosarcoma, liposarcoma)**	2	1.9
**Other or unclassifiable histologies**	16	15.1
**Total**	106	100

a Includes lymphoma, leukemia, multiple myeloma, Hodgkin disease, and monoclonal gammopathy of clinical significance.

Head and neck cancers constituted the largest group (29.2%, 31/106; 95% CI, 21.4%-38.5%). The larynx and pharynx (hypopharynx, pyriform sinus) were the most common subsites,^35^^,^^36^^,^^39^^,^^49^ followed by the oral cavity, tongue, tonsils,^50–52^ and lip.^26^ Although human papillomavirus (HPV) status is rarely reported in Bazex-associated cases, a few HPV-positive tumors have been documented.^35^^,^^50^ The male-to-female ratio in this subgroup is 6.75:1 (27:4).

Lung cancers formed the second-largest group at 20.8% (*n* = 22; 95% CI, 14.1%-29.4%), encompassing varied histologies: SCC,^20^^,^^53^ adenocarcinoma,^40^^,^^47^ small cell carcinoma,^11^ and bronchial carcinoid.^54^ Notably, one lung adenocarcinoma demonstrated high programmed death-ligand 1 (PD-L1) expression (>90%) without EGFR/anaplastic lymphoma kinase mutations.^47^ Seven of 22 patients with lung cancer (31.8%) were women.

Gastrointestinal malignancies comprised 20.7% (*n* = 22; 95% CI, 14.1%-29.4%) of cases. Esophageal tumors (11.3%, *n* = 12) were predominantly SCC,^1^^,^^38^^,^^55–60^ alongside occasional adenocarcinomas and other non-squamous types.^31^^,^^48^^,^^61^ The remaining GI cancers made up (9.4%, *n* = 10) included gastric adenocarcinoma (such as linitis plastica),^30^ colorectal adenocarcinomas^12^^,^^13^^,^^29^ and pancreatic, hepatic, or biliary tract tumors.^33^^,^^43^^,^^62–65^

Genitourinary cancers (3.8%, *n* = 5), included prostate adenocarcinoma,^16^^,^^66^^,^^67^ bladder transitional cell carcinoma,^1^ and renal angiosarcoma.^68^ Gynecologic tumors (cervical SCC,^32^ serous ovarian carcinoma^34^) and breast cancers^14^^,^^15^ each represented 1.9% (*n* = 2).

Hematologic malignancies accounted for 8.5% (*n* = 9; 95% CI, 4.5%-15.4%), involving T-cell lymphoma,^17^^,^^18^ Hodgkin lymphoma,^69^^,^^70^ B-cell/follicular lymphoma,^71^^,^^72^ multiple myeloma,^73^ acute myeloid leukemia,^74^ and IgM monoclonal gammopathy.^75^ Rare presentations featured thymic carcinoma,^76^ thyroid carcinoma^77^ sarcoma,^19^ and neuroendocrine tumor.^78^ In 6.6% (*n* = 7) of cases, the primary tumor remained unidentified.^53^^,^^78–83^

#### Clinical staging and metastatic profile

Explicit tumor, node, metastasis (TNM) or equivalent staging data were available for 33.0% (35/106) of patients. When integrating documented advanced or metastatic courses that lacked formal TNM notation, broad stage information was inferable for 70.8% (75/106). Overall, the cohort skewed heavily toward late-stage disease at presentation.

Regional nodal involvement was prevalent, notably presenting as cervical lymphadenopathy (often bilateral, N2c) in patients with head and neck cases^28^^,^^35^^,^^50^^,^^66^ and mediastinal or hilar nodes in patients with lung cancers.^11^^,^^27^^,^^38^^,^^47^ The 7 patients (6.6%) lacking an identifiable primary tumor characteristically presented with isolated metastatic SCC in cervical or mediastinal nodes, consistent with carcinoma of unknown primary.^53^^,^^78–83^ Distant metastases primarily involved the lung, liver, and bone, with rare spread to the brain, ovary (Krukenberg tumor), or peritoneum^1^^,^^15^^,^^27^^,^^30^^,^^32^^,^^45^^,^^47^^,^^76^^,^^78^^,^^84^ (Table 2).

**Table 2.** Clinical staging distribution among 106 patients with Bazex syndrome.

Staging summary	*n*	%
**Explicit TNM/stage-equivalent available**	35	33.0
**Advanced/metastatic without formal TNM (inferred)**	40	37.7
**No staging information**	31	29.2
**Total with any stage information**	75	70.8
**Total**	106	100

The staged cases spanned a wide clinical range, from cT2N2cM0 HPV-positive tongue-base SCC^50^ and T4aN2cM0 laryngeal SCC^28^ to overtly metastatic esophageal SCC^60^.

### Dermatological manifestations and diagnostic features

#### Clinical morphology and disease evolution

Cutaneous lesions are typically described as symmetrical and psoriasiform, with erythematous-to-violaceous hyperkeratotic plaques on acral sites, which were frequently refractory to topical therapy.^28^^,^^38^^,^^58^ A violaceous hue was consistently noted on the hands, feet, ears, and nose.^36^^,^^41^^,^^85^ Palmar and plantar lesions occasionally presented as a diffuse yellow keratoderma with a honeycomb-like surface.^4^^,^^41^ In some patients, particularly Black patients, pronounced hyperpigmentation overshadow the classic violaceous erythema.^11^^,^^38^ Rare vesiculobullous presentations^85–87^ and systemic manifestations, such as carpal tunnel syndrome,^88^ were also reported.

In our dataset, cutaneous lesions on the hands and palms were explicitly documented in 62.3% (*n* = 66; 95% CI, 52.8%-70.9% rising to *n* = 78 with finger-only descriptions). Lesions on the feet/soles/toes were reported in 48.1% (*n* = 51; 95% CI, 38.8%-57.5%), ears (notably helices) in 26.4% (*n* = 28; 95% CI, 19.0%-35.5%), and the nose in 23.6% (*n* = 25; 95% CI, 16.5%-32.5%); nail involvement was documented in 73.6% (*n* = 78; 95% CI, 64.5%-81.0%). Ear lesions commonly began on the helices and extended further with disease progression.^36^^,^^81^^,^^89^

Cutaneous lesions progressed centripetally through 3 morphological stages.^8^^,^^90^ Stage 1 (acral phase) involved erythema and scaling limited to fingertips, toes, nasal bridge, and ear helices.^12^ Stage 2 (palmoplantar phase) involved extension to the palms and soles as keratoderma.^91^ Stage 3 (generalized phase) featured plaque spread to the elbows, knees, trunk, and scalp.

#### Nail manifestations and histopathology

Detailed analysis of the 78 patients with nail involvement revealed common changes including dystrophy, thickening, onycholysis, longitudinal ridging, yellow discoloration, and subungual hyperkeratosis. Among the 77 patients with nail involvement and reported chronology, nail changes preceded the cancer diagnosis in 83.1% (*n* = 64; 95% CI, 73.2%-89.9%), occurred concurrently in 7.8% (*n* = 6; 95% CI, 3.6%-16.0%), and followed the diagnosis in 9.1% (*n* = 7; 95% CI, 4.5%-17.6%). Notable manifestations included trachyonychia 10 months prior to the diagnosis of pulmonary SCC in 1 patient^92^ and onychomadesis in 3 patients.^41^^,^^45^^,^^56^ Compared with cutaneous lesions, nail changes improved more slowly and often persisted despite skin clearance.^29^^,^^72^^,^^87^ Recovery was gradual: while proximal nail growth could be documented by 11 weeks, a fully normal plate required over a year.^92^^,^^93^

Reported histopathologic findings were frequently nonspecific.^5^^,^^39^ Common epidermal findings included hyperkeratosis, parakeratosis, and acanthosis, occasionally accompanied by dyskeratotic keratinocytes or vacuolar interface change. ^1^^,^^4^^,^^20^^,^^27^^,^^50^ Dermal infiltrate variably contained eosinophils, neutrophils, and mononuclear cells, with rare Touton giant cells also reported.^1^^,^^86^^,^^94^ In bullous presentations, severe basal vacuolar degeneration lead to intraepidermal vesiculation or subepidermal clefting, with variable immunofluorescence findings.^11^^,^^86^^,^^87^

#### Chronological relationship and differential diagnosis

Cutaneous lesions appeared prior to the tumor diagnosis in 80.6% (83/103; 95% CI, 71.9%-87.1%)) of cases with a reported chronological relationship. Lesions were concurrent in 10.7% (*n* = 11/103; 95% CI, 6.1%-18.1%) and followed the diagnosis in 8.7% (*n* = 9/103; 95% CI, 4.7%-15.8%). Among the 76 patients whose cutaneous signs preceded the tumor with a quantifiable interval, the median lead-time was 5 months (IQR 2-10 months; range 3 weeks to 6 years). Patients with lung cancer demonstrated the longest lead times (averaging 10-18 months, with outliers at 2.5-3.5 years),^40^^,^^95^ whereas patients with head and neck cancers showed a wider range (2 months to 5 years).^4^^,^^42^ Patients with GI adenocarcinomas typically exhibited shorter intervals.^12^^,^^29^ The extent of cutaneous lesions often paralleled tumor progression: Stage 1 and 2 localized cutaneous lesions typically accompanied occult or locally advanced cancer,^12^^,^^36^^,^^91^ while Stage 3 generalized lesions frequently signaled heavy tumor burden or metastatic spread.^45^^,^^76^^,^^84^ Moreover, recurrence of cutaneous lesions consistently signaled tumor relapse prior to radiographic detection.^19^^,^^36^^,^^60^^,^^96^

Psoriasis was the most frequently reported initial misdiagnosis in the reviewed cases.^35^^,^^97^ To exclude differential diagnosis such as infections and connective tissue diseases, serological tests including Venereal Disease Research Laboratory test, rapid plasma reagin, and antinuclear antibody (ANA) panels were frequently utilized; however, rare malignancy-associated positive ANA titers were also reported.^11^^,^^14^^,^^33^^,^^60^ Furthermore, recent literature identified anti-programmed cell death protein 1 agents (e.g. pembrolizumab) as potential inducers of acral lesions resembling Bazex syndrome, necessitating careful clinicopathologic correlation to distinguish them from the true paraneoplastic dermatosis.^5^^,^^98^

### Therapeutic interventions and clinical outcomes

#### Treatment modalities and cutaneous response

Following oncological therapy, reported cutaneous outcomes included complete clearance in 36.8% (*n* = 39/106; 95% CI, 28.2%-46.3%) of patients, partial improvement in 30.2% (*n* = 32/106; 95% CI, 22.3%-39.5%), no change or worsening in 6.6% (*n* = 7/106), missing data in 15.1% (*n* = 16/106). Additionally, 11.3% (*n* = 12/106) of patients died before assessment. Among the 78 patients with an evaluable cutaneous response, a conservative response definition (complete or explicit partial improvement) yielded a 78.2% (*n* = 61/78; 95% CI, 67.8%-85.9%) response rate. The remaining 17 patients reported no change, worsening, or unclassifiable nonstandard responses.

Surgery yielded the most rapid improvement, with cutaneous changes noted within 48 hours and full clearance in 2-4 weeks,^12^^,^^42^^,^^49^^,^^89^ whereas chemotherapy or radiotherapy generally required over 6 weeks.^28^^,^^51^ Targeted therapies, such as gefitinib^27^ and pembrolizumab (in a tumor with PD-L1 > 90% expression),^47^ also resulted in lesion clearance. While cutaneous recurrence strongly correlated with ongoing or metastatic malignancy, in some cases, cutaneous lesions exhibited “clinical uncoupling,” where hyperkeratotic plaques persisted up to 2 years despite successful oncologic treatment.^23^^,^^58^^,^^76^

Topical monotherapies (corticosteroids, emollients) frequently failed while the malignancy remains active.^14^^,^^28^^,^^40^^,^^45^^,^^53^^,^^89^^,^^99^ However, palliative therapies offered symptomatic relief: 20% salicylic acid can reduce plantar keratoderma sufficiently to permit shoe wear,^4^^,^^87^ and systemic acitretin (25 mg/day) relieved severe burning and itching in conservatively managed malignancies (e.g. in-situ follicular lymphoma).^35^^,^^71^ Psoralen and ultraviolet A (PUVA) provided significant plaque clearance in chemotherapy-ineligible patients, although nail dystrophy remained unaffected.^72^^,^^87^ Additionally, 2% nitroglycerin ointment was utilized to prevent dry gangrene in necrotic digits.^50^ Systemic dexamethasone provided rapid palliative relief in end-of-life care,^100^ and in rare persistent cases, near-complete remission of cutaneous lesions was achieved with topical tretinoin 0.025%.^29^

#### Clinical outcomes and reported mortality

Terminal status was definitively reported for 95 patients (64 alive, 31 who died). Outcomes were not reported in 5 cases, 4 patients were lost to follow-up, and 2 were documented with recurrent disease without a final vital status. Reported clinical outcomes varied notably with the extent of cutaneous lesions: patients presenting with Stage 1 and 2 localized lesions frequently achieved durable remissions (1.5-6 years).^4^^,^^12^^,^^35^^,^^70^ Conversely, progression to Stage 3 generalized lesions was associated with rapid clinical deterioration and death, often within 1 year.^11^^,^^39^^,^^46^^,^^59^^,^^101^ Rapid deterioration was also observed in patients with advanced diseases receiving immunotherapy.^47^ Among the patients who died, metastatic complications were the predominant cause of death. Furthermore, patients with an unidentified occult primary carried an exceptionally poor prognosis. ^83^

To evaluate variables associated with reported mortality among the 95 evaluable patients, univariable analyses were performed. The presence of metastasis showed the strongest nominal association with reported mortality (25/58 vs 6/33; Fisher’s exact test, *P* = .021; *n *= 91, as 4 patients were excluded due to unreported metastatic status), followed by an aerodigestive primary site (24/58 vs 7/37; *P* = .026). However, neither association remained statistically significant after Bonferroni or Benjamini–Hochberg correction. Squamous cell carcinoma vs non-SCC histology (*P* = .279), male vs female sex (*P* = .227), chronology of cutaneous lesions (*P* = .782), and age (alive vs those who died: 63.9 ± 9.2 vs 64.2 ± 9.0 years; Mann–Whitney U, *P* = .896) were not associated with reported mortality.

## Discussion

Bazex syndrome is an obligate paraneoplastic dermatosis defined by symmetrical acral psoriasiform plaques with nail dystrophy.^5^^,^^21^ The classic profile from Bolognia’s 1991 review is a middle-aged Caucasian man with a smoking history whose cutaneous lesions herald an occult aerodigestive SCC.^6^ Our review of 106 cases provides a contemporary perspective on the syndrome’s published profile; the reported oncologic spectrum is broader and the demographic mix appears more diverse, suggesting that Bazex syndrome should be treated as a heterogeneous paraneoplastic condition, though these observations must be interpreted within the context of publication bias.

### Epidemiological and oncological considerations

Women made up a substantial proportion (27.4%) of our cohort, representing a notably higher percentage compared to 4%-7% reported in earlier reviews.^6^ Our data indicates a male-to-female ratio of 2.7:1, aligning more closely with recent estimates^4^ than the classic 22:1 ratio cited by Bolognia et al.^6^ Rather than indicating a genuine epidemiologic shift, this rise in reported cases among women likely reflects publication bias and growing recognition of the syndrome in gynecologic and breast cancers.^14^^,^^15^^,^^32^^,^^34^ Reported racial diversity is also broader; while historically associated with Caucasian individuals of European descent,^109^ our cohort demonstrates a global distribution. Clinicians should be aware that in African American patients, the violaceous erythema may present as hyperpigmentation.^11^^,^^17^

Concurrently, the documented oncologic spectrum extends well beyond the aerodigestive tract. The significant involvement of diverse non-aerodigestive malignancies suggests that traditional “aerodigestive” criteria should be broadened in clinical practice. Isolated reports linking the syndrome to sarcomas^19^^,^^68^ and thyroid carcinoma^77^ further highlight this expanding spectrum. This heterogeneous picture is further complicated by atypical cutaneous presentations that mimicked autoimmune blistering diseases in several patients^85–87^ or presented as rare pustular variants,^102^ underscoring that Bazex syndrome should be on the differential for any treatment-resistant acral dermatosis, regardless of classic risk factors.

### Pathogenesis

The exact pathophysiology of Bazex syndrome remains unknown; current mechanistic evidence derives largely from isolated clinical observations rather than established molecular pathways. Leading to several overlapping hypotheses. A Th2-polarized immune response has been proposed based on reports such as that by Amano et al.^20^, who observed dense Th2-cell infiltrates and strong EGFR expression in lesional keratinocytes in a patient with lung SCC. This led to the hypothesis that tumor-associated increases in IL-4, IL-5, IL-9, and IL-13 may suppress Th1 antitumor surveillance while promoting EGFR-mediated epidermal hyperproliferation.^20^^,^^21^

A second hypothesis involves tumor-derived growth factors, particularly TGF-α, and EGF, with possible additional contributions from insulin-like growth factor 1.^6^^,^^21^^,^^37^ TGF-α and EGF are thought to act through EGFR, and the reported improvement of cutaneous lesions with gefitinib has been cited as potential anecdotal support for EGFR pathway involvement.^27^ A third proposed mechanism is molecular mimicry between tumor neoantigens and epidermal or basement-membrane components; this has been postulated due to variable immunofluorescence findings, including IgG, IgA, IgM, and C3 deposition along the dermal–epidermal junction in some patients.^11^^,^^86^ Additional hypothetical contributors may include VEGF-mediated vascular signaling, as suggested by markedly elevated VEGF in an isolated report of a patient with concurrent Bazex syndrome and RS3PE,^47^ and nutritional factors such as zinc deficiency in selected patients with acrodermatitis-enteropathica-like features.^15^ These mechanisms are not mutually exclusive, and Bazex syndrome is hypothesized to reflect several converging tumor–host interactions rather than a single established pathway.

### Clinical implications, prognosis, and therapeutic strategies

Cutaneous lesions preceded cancer diagnosis in 80.6% of our cohort. This frequency is notably higher than Bolognia’s historical estimate^6^ of 67% and the approximately 50% reported in a more recent systematic review by Räßler et al.^5^ While the median lead time was 5 months, the temporal gap can be extraordinarily long in certain outliers, reaching up to 3.5 years before lung adenocarcinoma^40^ and 5 years before hypopharyngeal SCC.^42^ Exceptions exist, however, such as cutaneous lesions appearing only during tumor recurrence.^13^

Diagnosing Bazex syndrome remains challenging due to its clinical and histologic overlap with common dermatoses, particularly psoriasis. Authors frequently highlight that histology alone does not reliably distinguish the two; however, clinicopathologic discordance, together with dyskeratotic keratinocytes or vacuolar/interface changes, should prompt consideration of an underlying malignancy.^5^^,^^50^

In the reviewed literature, reported clinical outcomes appeared to depend primarily on the underlying cancer rather than the cutaneous disease itself. While metastatic disease and an aerodigestive primary site demonstrated the strongest nominal univariable associations with reported mortality, neither remained statistically significant after multiple-comparison correction. Variables such as sex, SCC histology, chronology of cutaneous lesions, and age also showed no significant association with reported mortality. These findings suggest that advanced tumor burden likely drives clinical decline, but any definitive conclusions regarding variables associated with reported mortality remain limited by small sample size and missing data.

In the reported cases, definitive treatment of the cutaneous lesions relied on the eradication of the underlying malignancy,^5^^,^^6^ though complete clearance was achieved in only a subset of patients in our analysis. Given the EGFR overexpression observed by Amano et al.^20^, targeted inhibitors like gefitinib offer a viable second-line option for corticosteroid-refractory disease.^27^ When the tumor cannot be resected, systemic retinoids (acitretin, etretinate)^35^^,^^103^ and PUVA^72^^,^^87^ may be useful palliative options. Interestingly, pembrolizumab presents a clinical paradox: it relieved both the cutaneous lesions and concurrent RS3PE in a patient with metastatic lung adenocarcinoma,^47^ while O’Keeffe et al.^98^ documented a psoriasiform flare during pembrolizumab therapy, highlighting the difficulty of distinguishing Bazex syndrome from checkpoint-inhibitor toxicity.

## Limitations

Our review inherently carries the limitations of case report literature, primarily publication bias, which favors atypical presentations and may skew the clinical profile toward rare associations. Crucially, our mortality analysis must be interpreted with caution due to highly variable follow-up durations and the retrospective nature of the data. Reporting quality was uneven across the decades: missing data regarding terminal vital status and metastatic involvement restricted our comparative analyses. Similarly, explicit TNM staging was often unavailable in older reports, requiring us to infer broader disease stages based on clinical descriptions.

Diagnostic and reporting inconsistencies further constrain our findings. Standardized diagnostic criteria were not uniformly applied historically, and our figures likely underestimate true ear and nasal involvement because many source texts relied on nonspecific “acral” terminology. Finally, restricting our search to English-language publications introduces potential language bias especially given the historical and international scope of Bazex syndrome literature, and despite meticulous cross-referencing of demographic and institutional data, the theoretical risk of duplicate patient reporting across decades cannot be entirely eliminated. To mitigate these limitations and facilitate future meta-analytic pooling, full patient-level data are provided in Table S1.

## Conclusion

Bazex syndrome presents a significantly broader oncologic and demographic spectrum in contemporary literature than its classical definition suggests. While it remains a hallmark cutaneous marker for head and neck SCC, its documented associations now span a diverse array of malignancies, including various adenocarcinomas, hematologic neoplasms, and rare mesenchymal tumors, across both sexes and diverse ethnicities. For clinicians, these findings highlight an important diagnostic consideration: any atypical, treatment-resistant acral psoriasiform lesions should prompt a thorough search for an occult malignancy. Moving forward, the establishment of prospective registries using standardized diagnostic criteria will be essential to elucidate the true epidemiology, natural history, and optimal management of Bazex syndrome.

## Supplementary Material

oyag376_Supplementary_Data

## Data Availability

All extracted case-level data analyzed during this study are included in this published article and its supplementary information files (Table S1).
