# Supplementary material for: Acrokeratosis paraneoplastica (Bazex syndrome): a systematic review and quantitative analysis of 106 cases
Source: Oncologist. 2026 Sep 24;31(11):oyag376. doi: 10.1093/oncolo/oyag376 (PMC13650690; doi:10.1093/oncolo/oyag376)
Supplement: Supplementary file 1 [file oyag376_supplementary_data.docx]

**Supplementary Table S1**

Detailed case-level characteristics of 106 patients with Bazex syndrome, sorted by manuscript reference order.

| **Supplementary Case No.** | **Paper Ref. No.** | **Citation** | **Study Type** | **Age / Sex** | **Risk Factors / Comorbidities** | **Underlying Malignancy** | **Chronology** | **Cutaneous Findings** | **Histopathology** | **Treatment** | **Response / Outcome** |
| --- | --- | --- | --- | --- | --- | --- | --- | --- | --- | --- | --- |
| 1 | 1 | [1] Arregui et al. (1993) | Case series | 41 / Male | Weight loss | Site: Esophagus; Histology: Squamous cell carcinoma (SCC); TNM/Stage: Advanced (metastatic); Metastasis: Yes | Before (11 months) | Distribution: Fingers, ears; Nails: Onychodystrophy | Acanthosis | Radiotherapy | Skin response: Partial improvement; Outcome: Lost to follow-up |
| 2 | 1 | [1] Arregui et al. (1993) | Case series | 73 / Male | History of bladder cancer | Site: Bladder; Histology: Transitional cell carcinoma; TNM/Stage: Not reported; Metastasis: No | Before | Distribution: Fingers, toes; Nails: Nail shedding | Hyperkeratosis | Mitomycin C | Skin response: Complete resolution; Outcome: Alive |
| 3 | 4 | [4] Yamada et al. (2024) | Case series | 78 / Male | History of prostate cancer | Site: Larynx (glottis); Histology: Squamous cell carcinoma (SCC); TNM/Stage: Not reported; Metastasis: No | Before (11 months) | Distribution: Palms, soles; Nails: Not reported | eosinophilic masses, hyperkeratosis, acanthosis, and perivascular lymphocyte infiltration | Radiotherapy | Skin response: Complete resolution; Outcome: Alive |
| 4 | 4 | [4] Yamada et al. (2024) | Case series | 84 / Male | Smoker | Site: Lung; Histology: Squamous cell carcinoma (SCC); TNM/Stage: Advanced (metastatic); Metastasis: Yes | Before (1 month) | Distribution: Soles (honeycomb); Nails: Not reported | Keratin pearls, arterial invasion | Not reported | Skin response: Not reported; Outcome: Died |
| 5 | 9 | [9] Rio Ramirez (2007) | Case report | 78 / Male | Pulmonary fibrosis | Site: Lung; Histology: Adenocarcinoma; TNM/Stage: Not reported; Metastasis: No | Before (4 months) | Distribution: Hands, feet; Nails: Nail thickening | Not reported | Palliative care | Skin response: Died before resolution; Outcome: Died |
| 6 | 10 | [10] O'Brien (1995) | Case report | 67 / Female | Chronic obstructive pulmonary disease | Site: Oropharynx; Histology: Squamous cell carcinoma (SCC); TNM/Stage: Not reported; Metastasis: No | Before (2 years) | Distribution: Fingers, nose; Nails: Loss of nail plate | Psoriasiform changes | Chemotherapy + PUVA | Skin response: Partial improvement; Outcome: Died |
| 7 | 11 | [11] Zarzour et al. (2011) | Case report | 58 / Female | Systemic lupus erythematosus | Site: Lung; Histology: Small cell carcinoma; TNM/Stage: Advanced (metastatic); Metastasis: Yes | Before (2 years) | Distribution: Arms, legs, nose; Nails: Brittle, atrophied nails | IgG/C3 at basement membrane zone | Palliative care | Skin response: No change; Outcome: Died |
| 8 | 12 | [12] Hsu et al. (2000) | Case report | 63 / Male | Alcohol use | Site: Colon; Histology: Adenocarcinoma; TNM/Stage: Dukes B1; Metastasis: No | Before (1 month) | Distribution: Palms, soles; Nails: Nail dystrophy | Exocytosis | Resection | Skin response: Complete resolution; Outcome: Alive |
| 9 | 13 | [13] Tay & Pang (2014) | Case report | 76 / Female | History of colon cancer | Site: Colon; Histology: Adenocarcinoma; TNM/Stage: Advanced (metastatic); Metastasis: Yes | After (6 months) | Distribution: Hands, feet, ears; Nails: Onycholysis | Vacuolar alteration | Radiotherapy | Skin response: No change; Outcome: Alive |
| 10 | 14 | [14] Akhyani et al. (2004) | Case report | 62 / Female | Hypertension, hypertriglyceridaemia, osteoarthritis | Site: Breast; Histology: Infiltrating ductal carcinoma; TNM/Stage: Stage IIA; Metastasis: No | Before (8 months) | Distribution: Soles, hands; Nails: Not reported | Psoriasiform changes, vacuolar degeneration | Mastectomy + chemotherapy (CMF regimen) | Skin response: Partial improvement; Outcome: Alive |
| 11 | 15 | [15] Taher et al. (2007) | Case report | 68 / Female | History of breast cancer | Site: Breast; Histology: Lobular carcinoma; TNM/Stage: Advanced (metastatic); Metastasis: Yes | Concurrent | Distribution: Face, hands; Nails: Nail dystrophy | Dyskeratosis | Chemotherapy (docetaxel) | Skin response: Partial improvement; Outcome: Alive |
| 12 | 16 | [16] Da Rosa et al. (2009) | Case report | 54 / Male | Systemic lupus erythematosus | Site: Prostate; Histology: Adenocarcinoma; TNM/Stage: Gleason 6 (3+3); Metastasis: No | Before (1 year) | Distribution: Hands, feet, knees; Nails: Vertical ridging | Psoriasiform changes | Prostatectomy | Skin response: Partial improvement; Outcome: Alive |
| 13 | 17 | [17] McClatchey et al. (2019) | Case report | 56 / Male | Pruritus | Site: Hematologic; Histology: T-cell lymphoma; TNM/Stage: Advanced (metastatic); Metastasis: Yes | Before (6 years) | Distribution: Arms, legs, face; Nails: Not reported | Spongiosis | Chemotherapy | Skin response: Partial improvement; Outcome: Died |
| 14 | 18 | [18] Lin et al. (2001) | Case report | 57 / Female | Cervical lymphadenopathy | Site: Hematologic; Histology: T-cell lymphoma; TNM/Stage: Stage IV; Metastasis: Yes | Concurrent | Distribution: Palms, soles; Nails: Not reported | Not reported | Chemotherapy | Skin response: Minimal improvement; Outcome: Died |
| 15 | 19 | [19] Sator et al. (2006) | Case report | 71 / Male | Liposarcoma | Site: Retroperitoneum; Histology: Liposarcoma; TNM/Stage: Advanced (metastatic); Metastasis: Yes | After (6 weeks) | Distribution: Ears, knuckles; Nails: Yellow discoloration | Cytoid bodies | Surgery + radiotherapy | Skin response: Partial improvement; Outcome: Recurred |
| 16 | 20 | [20] Amano et al. (2016) | Case report | 82 / Male | History of gastric cancer | Site: Lung; Histology: Squamous cell carcinoma (SCC); TNM/Stage: Not reported; Metastasis: No | Before (6 months) | Distribution: Palms, soles; Nails: Not reported | Parakeratosis | Lobectomy | Skin response: Complete resolution; Outcome: Alive |
| 17 | 22 | [22] Khachemoune (2004) | Case report | 70 / Male | Smoker(1 pack per day), alcohol use | Site: Larynx; Histology: Squamous cell carcinoma (SCC); TNM/Stage: Stage IV; Metastasis: Yes | Before (3 months) | Distribution: Palms, soles; Nails: Yellow discoloration, dystrophy | Psoriasiform changes | Surgery + chemotherapy | Skin response: Complete resolution; Outcome: Alive |
| 18 | 23 | [23] Fasanmade et al. (2009) | Case report | 60 / Male | Non-smoker | Site: Oropharynx; Histology: Squamous cell carcinoma (tonsil); TNM/Stage: Stage IVA (T4N1); Metastasis: Yes | Concurrent | Distribution: Hand (hypothenar); Nails: Not reported | Not reported | Neck dissection + radiotherapy | Skin response: No change; Outcome: Alive |
| 19 | 26 | [26] Gaurav & Grover (2022) | Case report | 40 / Male | Smoker (15 pack-years) | Site: Lip (lower); Histology: Squamous cell carcinoma (SCC); TNM/Stage: Stage III (T3N1M0); Metastasis: Yes | Before (6 months) | Distribution: Ears, palms, soles; Nails: Xanthonychia, chloronychia | Dyskeratosis | Surgery (planned) | Skin response: Not reported; Outcome: Lost to follow-up |
| 20 | 27 | [27] Zhao et al. (2016) | Case report | 83 / Male | Smoker | Site: Lung; Histology: Adenocarcinoma; TNM/Stage: Advanced (metastatic); Metastasis: Yes | Before (5 years) | Distribution: Nose, ears, knees; Nails: Yellow-brown nails | Hyperkeratosis | Gefitinib | Skin response: Partial improvement; Outcome: Alive |
| 21 | 28 | [28] Shikino & Ikusaka (2017) | Case report | 84 / Male | Smoker | Site: Larynx; Histology: Squamous cell carcinoma (SCC); TNM/Stage: Stage IVA; Metastasis: Yes | Before (2 months) | Distribution: Hands, feet, ears; Nails: Yellow discoloration, detachment | Not reported | Radiotherapy | Skin response: Complete resolution; Outcome: Alive |
| 22 | 29 | [29] Baek et al. (2008) | Case report | 63 / Female | History of tuberculosis | Site: Colon; Histology: Adenocarcinoma; TNM/Stage: Stage III (T3N1M0); Metastasis: Yes | Before (1 month) | Distribution: Palms, soles; Nails: Onycholysis | Vacuolar degeneration | Surgery + topical tretinoin | Skin response: Complete resolution; Outcome: Alive |
| 23 | 30 | [30] Robert et al. (2014) | Case report | 73 / Female | Smoker | Site: Stomach; Histology: Gastric linitis plastica; TNM/Stage: Advanced (metastatic); Metastasis: Yes | Before (2 months) | Distribution: Hands, feet; Nails: Abnormalities present | Not reported | Chemotherapy (FOLFOX) | Skin response: Partial (then worsened); Outcome: Died |
| 24 | 31 | [31] Zhang (2025) | Case report | 70s / Female | Not reported | Site: Esophagus; Histology: Adenocarcinoma; TNM/Stage: Advanced (metastatic); Metastasis: Yes | Before (8 months) | Distribution: Ears, face, hands, feet, buttocks; Nails: Thickened, discolored | Hyperkeratosis, parakeratosis | Palliative care | Skin response: Worsened; Outcome: Died |
| 25 | 32 | [32] Hempen et al. (2015) | Case report | 60 / Female | Pancreatitis | Site: Ovary; Histology: Serous carcinoma; TNM/Stage: Advanced (metastatic); Metastasis: Yes | Concurrent | Distribution: Plantar feet; Nails: Not reported | Hyperkeratosis | Chemotherapy (carboplatin/paclitaxel) | Skin response: Partial regression; Outcome: Alive |
| 26 | 33 | [33] Karabulut et al. (2006) | Case report | 57 / Female | Weight loss | Site: Biliary tract; Histology: Cholangiocarcinoma; TNM/Stage: Not reported; Metastasis: No | Before (2 months) | Distribution: Face, ears; Nails: Nail ridging | Hyperkeratosis | Not reported | Skin response: Not reported; Outcome: Lost to follow-up |
| 27 | 34 | [34] Squires et al. (2016) | Case report | 56 / Female | Not reported | Site: Cervix; Histology: Squamous cell carcinoma (SCC); TNM/Stage: FIGO IIB; Metastasis: No | Before (4 months) | Distribution: Hands, feet, back; Nails: Pitting | Hyperkeratosis | Chemoradiotherapy | Skin response: Complete resolution; Outcome: Alive |
| 28 | 35 | [35] Eckstein et al. (2020) | Case series | 60 / Male | Smoker(80 pack-year), alcohol use | Site: Hypopharynx; Histology: Squamous cell carcinoma (SCC); TNM/Stage: Stage IVA (cT2N2cM0); Metastasis: Yes | Concurrent | Distribution: Hands, feet, ears; Nails: Dryness, flaking, and thickening of fingernails | Hyperkeratosis, horn cysts with focal intracorneal acute inflammation, psoriaform dermatitis | Chemoradiotherapy + acitretin | Skin response: Complete resolution; Outcome: Alive |
| 29 | 35 | [35] Eckstein et al. (2020) | Case series | 55 / Female | Smoker | Site: Oropharynx; Histology: Squamous cell carcinoma (SCC); TNM/Stage: Stage IVA (cT2N2cM0); Metastasis: Yes | Before (6 months) | Distribution: Hands, feet; Nails: Brittle nails, nail plate lifting | Not reported | Chemoradiotherapy | Skin response: Partial improvement; Outcome: Alive |
| 30 | 35 | [35] Eckstein et al. (2020) | Case series | 48 / Male | Smoker | Site: Hypopharynx; Histology: Squamous cell carcinoma (SCC); TNM/Stage: Stage IVA (cT2N2cM0); Metastasis: Yes | After (1 month) | Distribution: Palms, soles, shins; Nails: Not reported | Not reported | Chemoradiotherapy | Skin response: Partial improvement; Outcome: Died |
| 31 | 36 | [36] Mounsey & Brown (1992) | Case report | 66 / Male | Smoker | Site: Larynx; Histology: Squamous cell carcinoma (recurrent); TNM/Stage: T1N0M0; Metastasis: Yes | Concurrent | Distribution: Hands, feet; Nails: Nail ridging | Hyperkeratosis | Palliative care | Skin response: Not reported; Outcome: Not reported |
| 32 | 37 | [37] Bazex et al. (1994) | Case report | 54 / Male | Smoker | Site: Lung; Histology: Squamous cell carcinoma + small cell carcinoma; TNM/Stage: Advanced (metastatic); Metastasis: Yes | Before (5 months) | Distribution: Face, nose; Nails: Nails spared | Parakeratosis | Chemotherapy | Skin response: Complete resolution; Outcome: Alive |
| 33 | 38 | [38] Poligone et al. (2007) | Case report | 62 / Male | Alcohol use | Site: Esophagus; Histology: Squamous cell carcinoma (SCC); TNM/Stage: Stage III; Metastasis: No | Before (2 months) | Distribution: Ears, hands; Nails: Nail thickening | Necrotic keratinocytes | Chemoradiotherapy | Skin response: Complete resolution; Outcome: Alive |
| 34 | 39 | [39] Ben-Hassen et al. (2018) | Case report | 62 / Male | Smoker | Site: Larynx; Histology: Squamous cell carcinoma (SCC); TNM/Stage: Advanced (metastatic); Metastasis: Yes | Before (months) | Distribution: Ears, hands, feet; Nails: Yellow hyperkeratosis | Hyperkeratosis | Chemotherapy | Skin response: Not reported; Outcome: Died |
| 35 | 40 | [40] Crucitti et al. (2009) | Case report | 55 / Male | Smoker | Site: Lung; Histology: Adenocarcinoma; TNM/Stage: Stage I (T2N0M0); Metastasis: No | Before (3.5 years) | Distribution: Hands, feet; Nails: Yellow discoloration, dystrophy | Not reported | Lobectomy+cisplatin and navelbine | Skin response: Complete resolution; Outcome: Alive |
| 36 | 41 | [41] Valdivielso et al. (2005) | Case report | 64 / Male | Cirrhosis | Site: Lung; Histology: Squamous cell carcinoma (SCC); TNM/Stage: Stage IIIA (T2N2M0); Metastasis: Yes | Before (2 months) | Distribution: Fingers, nose; Nails: Onychomadesis | Orthokeratosis | Chemotherapy + radiotherapy | Skin response: Partial response (then worsened); Outcome: Died |
| 37 | 42 | [42] Sarkar et al. (1998) | Case report | 61 / Male | Alcohol use | Site: Hypopharynx; Histology: Squamous cell carcinoma (SCC); TNM/Stage: T3N0M0; Metastasis: No | Before (5 years) | Distribution: Hands, ears; Nails: Nail dystrophy, yellowish discoloration, periungual swelling | Not reported | Laryngectomy | Skin response: Not reported; Outcome: Not reported |
| 38 | 43 | [43] Holzgruber et al. (2022) | Case report | 69 / Male | Cirrhosis | Site: Liver; Histology: Hepatocellular carcinoma; TNM/Stage: Not reported; Metastasis: No | After | Distribution: Hands, legs; Nails: Not reported | Nonspecific | Sorafenib (palliative) | Skin response: No change; Outcome: Lost to follow-up |
| 39 | 44 | [44] Koh et al. (2012) | Case report | 61 / Male | Stroke | Site: Hypopharynx; Histology: Squamous cell carcinoma (SCC); TNM/Stage: Advanced (metastatic); Metastasis: Yes | Concurrent | Distribution: Scalp, ears, nose; Nails: Nails unaffected | Not reported | Palliative radiotherapy | Skin response: Not reported; Outcome: Alive |
| 40 | 45 | [45] Graves et al. (2014) | Case report | 60s / Male | Not reported | Site: Larynx; Histology: Basaloid squamous cell carcinoma; TNM/Stage: Stage IV (metastatic); Metastasis: Yes | Before (9 months) | Distribution: Chest, back, penis; Nails: Onychomadesis | Psoriasiform changes | Palliative care | Skin response: No change; Outcome: Alive |
| 41 | 46 | [46] Humphrey et al. (2015) | Case report | 57 / Male | Weight loss | Site: Esophagus; Histology: Epithelioid neoplasm; TNM/Stage: Not reported; Metastasis: No | Concurrent | Distribution: Hands, feet, nose; Nails: Not reported | Interface dermatitis | Chemoradiotherapy | Skin response: Died before resolution; Outcome: Died |
| 42 | 47 | [47] Aoshima et al. (2019) | Case report | 69 / Male | Diabetes mellitus | Site: Lung; Histology: Adenocarcinoma; TNM/Stage: Stage IVB; Metastasis: Yes | Before (6 months) | Distribution: Fingers, ears; Nails: Not reported | Hyperkeratosis | Pembrolizumab | Skin response: Partial improvement; Outcome: Alive |
| 43 | 48 | [48] Hagedorn (1983) | Case report | 67 / Male | Alcohol use | Site: Esophagus; Histology: Carcinoma; TNM/Stage: Not reported; Metastasis: No | After (9 months) | Distribution: Nose, ears; Nails: Nail ridging | Capillaritis | Radiotherapy | Skin response: Partial improvement; Outcome: Alive |
| 44 | 49 | [49] Couselo-Rodríguez (2024) | Case report | 52 / Female | Ex-smoker | Site: Larynx; Histology: Epidermoid carcinoma; TNM/Stage: Advanced (metastatic); Metastasis: Yes | Not reported | Distribution: Fingers, feet, cheeks; Nails: Not reported | Not reported | Laryngectomy + radiotherapy | Skin response: Complete resolution; Outcome: Alive |
| 45 | 50 | [50] Adelman et al. (2018) | Case report | 56 / Female | Smoker | Site: Oropharynx; Histology: Squamous cell carcinoma (HPV-positive); TNM/Stage: Stage IVA (T2N2cM0); Metastasis: Yes | Before (2 months) | Distribution: Hands, fingers, toes; Nails: Ridging, nail plate lifting | Spongiosis, dyskeratosis | Chemoradiotherapy | Skin response: Near resolution; Outcome: Alive |
| 46 | 51 | [51] Buxtorf et al. (2001) | Case report | 58 / Male | Ex-smoker | Site: Tongue; Histology: Squamous cell carcinoma (SCC); TNM/Stage: T2N1M0; Metastasis: Yes | Before (4 months) | Distribution: Hands, nose; Nails: Hyperkeratosis | Not reported | Chemotherapy + radiotherapy | Skin response: Complete resolution; Outcome: Alive |
| 47 | 52 | [52] Santos-Silva (2010) | Case report | 61 / Male | History of tongue cancer | Site: Mediastinum; Histology: Unknown (metastatic); TNM/Stage: T2N0M0; Metastasis: No | After | Distribution: Hands, legs, nose; Nails: Nail thickening | Dyskeratosis | Palliative care | Skin response: Died before resolution; Outcome: Died |
| 48 | 53 | [53] Handfield-Jones (1992) | Case series | 73 / Female | Smoker | Site: Lung; Histology: Squamous cell carcinoma (SCC); TNM/Stage: Advanced (metastatic); Metastasis: Yes | Before (2 months) | Distribution: Fingers, toes; Nails: Nail ridging | Psoriasiform changes | Radiotherapy | Skin response: Partial improvement; Outcome: Alive |
| 49 | 53 | [53] Handfield-Jones (1992) | Case series | 65 / Male | Not reported | Site: Unknown primary; Histology: Anaplastic carcinoma; TNM/Stage: Advanced (metastatic); Metastasis: Yes | Before (3 months) | Distribution: Toes only; Nails: Nail dystrophy | Psoriasiform changes | Radiotherapy | Skin response: Partial improvement; Outcome: Alive |
| 50 | 53 | [53] Handfield-Jones (1992) | Case series | 73 / Male | Bronchitis | Site: Lung; Histology: Squamous cell carcinoma (SCC); TNM/Stage: Advanced (metastatic); Metastasis: Yes | Before (2 months) | Distribution: Hands, feet; Nails: Flaking | Psoriasiform changes | Corticosteroids | Skin response: Died before resolution; Outcome: Died |
| 51 | 54 | [54] Brenner et al. (1987) | Case report | 50 / Male | Smoker | Site: Lung; Histology: Bronchial carcinoid; TNM/Stage: Not reported; Metastasis: No | Concurrent | Distribution: Elbows, knees; Nails: Nail dystrophy | Psoriasiform changes | Pneumonectomy | Skin response: Complete resolution; Outcome: Alive |
| 52 | 55 | [55] Grimwood & Lekan (1987) | Case report | 67 / Female | Not reported | Site: Esophagus; Histology: Squamous cell carcinoma (SCC); TNM/Stage: Not reported; Metastasis: No | Before (6 months) | Distribution: Hands, scalp; Nails: Nail dystrophy | Spongiosis | Resection | Skin response: Complete resolution; Outcome: Alive |
| 53 | 56 | [56] Rodrigues et al. (2013) | Case report | 73 / Female | Smoker | Site: Esophagus; Histology: Squamous cell carcinoma (SCC); TNM/Stage: Not reported; Metastasis: No | Before (40 days) | Distribution: Hands, nose, ears; Nails: Onychomadesis | Psoriasiform changes | Not reported | Skin response: Died before resolution; Outcome: Died |
| 54 | 57 | [57] Bundela et al. (2022) | Case report | 52 / Male | Weight loss | Site: Esophagus; Histology: Squamous cell carcinoma (SCC); TNM/Stage: Not reported; Metastasis: No | Concurrent (3 months) | Distribution: Hands, palms, soles; Nails: Nail dystrophy | Hyperkeratosis | Chemoradiotherapy | Skin response: Marked improvement; Outcome: Alive |
| 55 | 58 | [58] Viteri et al. (2005) | Case report | 51 / Male | Smoker | Site: Esophagus; Histology: Squamous cell carcinoma (SCC); TNM/Stage: Advanced (metastatic); Metastasis: Yes | Before | Distribution: Palms, soles; Nails: Nail detachment | Not reported | Not reported | Skin response: Died before resolution; Outcome: Died |
| 56 | 59 | [59] Louvel et al. (2008) | Case report | 55 / Male | Alcohol use | Site: Esophagus; Histology: Squamous cell carcinoma (SCC); TNM/Stage: Advanced (metastatic); Metastasis: Yes | Before (4 months) | Distribution: Nose, toes, hands; Nails: Ungual involvement | Nonspecific | Chemotherapy (cisplatin) | Skin response: Complete resolution; Outcome: Died |
| 57 | 60 | [60] Medenica et al. (2008) | Case report | 50 / Male | Psoriasis | Site: Esophagus; Histology: Squamous cell carcinoma (SCC); TNM/Stage: Stage II (T2N0Mx); Metastasis: No | Before (3 months) | Distribution: Nose, ears, hands; Nails: Subungual hyperkeratosis | Spongiosis | Resection | Skin response: Complete resolution; Outcome: Alive |
| 58 | 61 | [61] Douglas et al. (1991) | Case report | 82 / Male | Dysphagia | Site: Esophagus; Histology: Adenocarcinoma; TNM/Stage: Advanced (metastatic); Metastasis: Yes | Before (2 years) | Distribution: Fingers, ears; Nails: Longitudinal ridging + distal thickening + destructive nail dystrophy | Hyperkeratosis | Palliative care | Skin response: Worsened; Outcome: Died |
| 59 | 62 | [62] Iwanami et al. (2018) | Case report | 76 / Female | Not reported | Site: Pancreas; Histology: Adenocarcinoma; TNM/Stage: Advanced (metastatic); Metastasis: Yes | Before (3 months) | Distribution: Distal extremities; Nails: Not reported | Not reported | Not reported | Skin response: Not reported; Outcome: Alive |
| 60 | 63 | [63] Narasimha et al. (2020) | Case report | 64 / Female | Hypertension | Site: Pancreas; Histology: Adenocarcinoma; TNM/Stage: Not reported; Metastasis: No | Before (6 months) | Distribution: Extremities, abdomen; Nails: Not reported | Psoriasiform changes | Chemotherapy (FOLFIRINOX) | Skin response: Not reported; Outcome: Alive |
| 61 | 64 | [64] Horton et al. (2020) | Case report | 74 / Male | Hypertension | Site: Pancreas; Histology: Adenocarcinoma; TNM/Stage: Advanced (metastatic); Metastasis: Yes | Before (3 weeks) | Distribution: Ears, hands, feet; Nails: Thickened nails | Psoriasiform changes | Palliative care | Skin response: Not reported; Outcome: Alive |
| 62 | 65 | [65] Goto et al. (2011) | Case report | 75 / Male | Not reported | Site: Liver; Histology: Hepatocellular carcinoma; TNM/Stage: Not reported; Metastasis: No | Before (1 month) | Distribution: Face, palms; Nails: Dystrophic nails | Spongiosis | Hepatic resection | Skin response: Partial improvement; Outcome: Alive |
| 63 | 66 | [66] Figueroa-Silva (2017) | Case report | 72 / Male | History of prostate cancer | Site: Submandibular; Histology: Undifferentiated carcinoma; TNM/Stage: Advanced (metastatic); Metastasis: Yes | Before (1 year) | Distribution: Nose, fingers; Nails: Nail dystrophy | Parakeratosis | Chemotherapy + radiotherapy | Skin response: Minimal improvement; Outcome: Died |
| 64 | 67 | [67] Obasi & Garg (1987) | Case Report | 62 / Male | None reported (Non-smoker/drinker) | Site: Prostate; Histology: Adenocarcinoma; TNM/Stage: Advanced (metastatic); Metastasis: Yes | Before (6 yrs) | Distribution: Hands, feet, elbows, knees, buttocks; Nails: Nail dystrophy | Hyperkeratosis, acanthosis, papillomatosis, segmental hypergranulosis, dyskeratotic cells | Stilboestrol | Skin response: Partial improvement; Outcome: Alive |
| 65 | 68 | [68] Rubegni et al. (2014) | Case report | 75 / Female | Weight loss | Site: Kidney; Histology: Renal angiosarcoma; TNM/Stage: Not reported; Metastasis: No | Before | Distribution: Acral regions, trunk; Nails: Hyperkeratosis | Not reported | Nephrectomy | Skin response: Complete resolution; Outcome: Alive |
| 66 | 69 | [69] Lucker & Steijlen (1995) | Case report | 43 / Male | Anemia | Site: Hematologic; Histology: Hodgkin disease; TNM/Stage: Stage IIB (Hodgkin); Metastasis: Yes | Before (12 months) | Distribution: Fingers, nose; Nails: Onycholysis | Ichthyosis vulgaris | Chemotherapy | Skin response: Complete resolution; Outcome: Alive |
| 67 | 70 | [70] Nguyen et al. (2019) | Case report | 53 / Male | Weight loss | Site: Hematologic; Histology: Hodgkin lymphoma; TNM/Stage: Advanced (metastatic); Metastasis: Yes | Before (6 months) | Distribution: Face, palms, soles; Nails: Not reported | Nonspecific | Chemotherapy (ABVD) | Skin response: Complete resolution; Outcome: Alive |
| 68 | 71 | [71] Conde-Montero (2017) | Case report | 53 / Female | Smoker | Site: Hematologic; Histology: Follicular lymphoma; TNM/Stage: Not reported; Metastasis: No | Before (2 months) | Distribution: Soles, palms; Nails: Onychodystrophy | Hyperkeratosis | Acitretin | Skin response: Complete resolution; Outcome: Alive |
| 69 | 72 | [72] Abrou et al. (2007) | Case report | 63 / Male | Smoker | Site: Hematologic; Histology: B-cell lymphoma; TNM/Stage: Stage IIIA; Metastasis: No | Before (9 months) | Distribution: Hands, soles; Nails: Thickened nails, subungual debris | Hyperkeratosis | PUVA (palliative) | Skin response: Partial response (skin only); Outcome: Alive |
| 70 | 73 | [73] Kandemir Alibakan (2020) | Case report | 57 / Female | Anemia | Site: Hematologic; Histology: Multiple myeloma; TNM/Stage: Not reported; Metastasis: Not applicable | Not reported | Distribution: Hands, feet, trunk; Nails: Hyperkeratosis | Not reported | Bortezomib | Skin response: Complete resolution; Outcome: Alive |
| 71 | 74 | [74] Oka & Ono (2023) | Case report | 72 / Male | Not reported | Site: Hematologic; Histology: Acute myeloid leukemia; TNM/Stage: Not reported; Metastasis: Not applicable | Before (5 months) | Distribution: Hands, feet, back; Nails: Not reported | Parakeratosis | Induction chemotherapy | Skin response: Partial improvement; Outcome: Alive |
| 72 | 75 | [75] Faruqi et al. (2025) | Case report | 50s / Male | Recurrent cellulitis | Site: Hematologic; Histology: IgM monoclonal gammopathy; TNM/Stage: Not reported; Metastasis: Not applicable | Concurrent | Distribution: Plantar feet, hands; Nails: Not reported | Spongiosis, dyskeratosis | Bendamustine + rituximab | Skin response: Complete resolution; Outcome: Alive |
| 73 | 76 | [76] Chave et al. (2004) | Case report | 65 / Male | History of thymoma | Site: Thymus; Histology: Thymic carcinoma; TNM/Stage: Stage III; Metastasis: Yes | Before (2 months) | Distribution: Trunk, limbs; Nails: Thickened nails | Vacuolar degeneration | Palliative care | Skin response: Died before resolution; Outcome: Died |
| 74 | 77 | [77] Macca et al. (2022) | Case report | 62 / Male | History of thyroidectomy | Site: Thyroid; Histology: Papillary carcinoma; TNM/Stage: Stage IV (metastatic); Metastasis: Yes | After (12 months) | Distribution: Generalized; Nails: Subungual hyperkeratosis | Not reported | Palliative care | Skin response: Not reported; Outcome: Alive |
| 75 | 78 | [78] Halpern et al. (1995) | Case report | 70 / Male | Not reported | Site: Neuroendocrine; Histology: Metastatic malignancy; TNM/Stage: Advanced (metastatic); Metastasis: Yes | Not reported | Distribution: Digits, face; Nails: Not reported | Hyperkeratosis | Octreotide | Skin response: Complete resolution; Outcome: Alive |
| 76 | 79 | [79] Fleming et al. (2014) | Case report | 70 / Female | Smoker | Site: Unknown primary; Histology: Undifferentiated carcinoma; TNM/Stage: Advanced (metastatic); Metastasis: Yes | Before (8 months) | Distribution: Fingers, ears; Nails: Nail dystrophy (9 nails) | Dyskeratosis | Neck dissection + radiotherapy | Skin response: Not reported; Outcome: Alive |
| 77 | 80 | [80] Matsui et al. (2016) | Case report | 63 / Male | Hepatitis | Site: Unknown primary; Histology: Squamous cell carcinoma (cervical node); TNM/Stage: Advanced (metastatic); Metastasis: Yes | Before | Distribution: Face, hands, feet; Nails: Not reported | Not reported | Neck dissection | Skin response: Partial improvement; Outcome: Alive |
| 78 | 81 | [81] Wareing et al. (1996) | Case report | 71 / Male | Smoker | Site: Unknown primary; Histology: Squamous cell carcinoma (cervical); TNM/Stage: Advanced (metastatic); Metastasis: Yes | Before (18 months) | Distribution: Hands, nose; Nails: Nail ridging | Acanthosis, hydropic degeneration | Radiotherapy | Skin response: Partial improvement; Outcome: Alive |
| 79 | 82 | [82] Richard & Giroux (1987) | Case report | 55 / Male | Ulcer history | Site: Unknown primary; Histology: Squamous cell carcinoma (mediastinal); TNM/Stage: Advanced (metastatic); Metastasis: Yes | Before (4 months) | Distribution: Nose, ears; Nails: Thickened nails | Spongiosis | Radiotherapy | Skin response: Not reported; Outcome: Not reported |
| 80 | 83 | [83] Ljubenovic et al. (2009) | Case report | 56 / Male | Not reported | Site: Unknown; Histology: Anaplastic carcinoma; TNM/Stage: Advanced (metastatic); Metastasis: Yes | Before (1 month) | Distribution: Palms, soles, nose; Nails: Nail dystrophy | Hyperkeratosis | Palliative care | Skin response: Died before resolution; Outcome: Died |
| 81 | 84 | [84] Vatandoust et al. (2016) | Case report | 69 / Male | History of skin cancer | Site: Skin; Histology: Cutaneous squamous cell carcinoma; TNM/Stage: Advanced (metastatic); Metastasis: Yes | Before (18 months) | Distribution: Acral, symmetrical; Nails: Nail dystrophy | Not reported | Chemotherapy (cisplatin/5-fluorouracil) | Skin response: Complete resolution; Outcome: Alive |
| 82 | 85 | [85] Duran-Vian et al. (2020) | Case report | 57 / Male | Alcohol use | Site: Oropharynx; Histology: Squamous cell carcinoma (SCC); TNM/Stage: Not reported; Metastasis: No | Before (3 months) | Distribution: Hands, feet, ears, trunk (bullae); Nails: Not reported | Interface dermatitis, blisters | Chemotherapy | Skin response: Partial improvement (then died); Outcome: Died |
| 83 | 86 | [86] Mutasim & Meiri (1999) | Case report | 67 / Male | History of prostate cancer | Site: Larynx; Histology: Squamous cell carcinoma (SCC); TNM/Stage: Not reported; Metastasis: No | Before (3 weeks) | Distribution: Hands, knees; Nails: Nail dystrophy | Intraepidermal vesicles | Laryngectomy | Skin response: Partial improvement; Outcome: Alive |
| 84 | 87 | [87] Gill et al. (2001) | Case report | 59 / Male | Smoker | Site: Hypopharynx; Histology: Squamous cell carcinoma (SCC); TNM/Stage: Advanced (metastatic); Metastasis: Yes | Before (4 months) | Distribution: Hands, nose; Nails: Nail dystrophy | Microvesicles | Surgery + PUVA | Skin response: Complete resolution; Outcome: Alive |
| 85 | 88 | [88] Poskitt & Duffill (1992) | Case report | 70 / Male | History of esophageal cancer | Site: Oropharynx; Histology: Squamous cell carcinoma (SCC); TNM/Stage: Advanced (metastatic); Metastasis: Yes | Before (8 months) | Distribution: Nose, ears; Nails: Nail ridging | Acanthosis | Radiotherapy | Skin response: Partial improvement; Outcome: Died |
| 86 | 89 | [89] Miquel et al. (1997) | Case report | 64 / Male | Smoker | Site: Larynx (epiglottis); Histology: Squamous cell carcinoma (SCC); TNM/Stage: Advanced (metastatic); Metastasis: Yes | Before (8 months) | Distribution: Hands, knees; Nails: Nail dystrophy | Orthokeratosis | Laryngectomy | Skin response: Complete resolution; Outcome: Recurred |
| 87 | 90 | [90] Laccourreye (1996) | Case report | 62 / Male | Dysphagia | Site: Hypopharynx; Histology: Squamous cell carcinoma (SCC); TNM/Stage: Advanced (metastatic); Metastasis: Yes | Before (6 months) | Distribution: Fingers, toes; Nails: Nail thickening | Not performed | Palliative radiotherapy | Skin response: Died before resolution; Outcome: Died |
| 88 | 91 | [91] Lechien et al. (2017) | Case report | 62 / Male | Tongue pain | Site: Tongue; Histology: Squamous cell carcinoma (SCC); TNM/Stage: Stage IV; Metastasis: Yes | Before | Distribution: Generalized; Nails: Not reported | Findings consistent with Bazex syndrome | Chemotherapy | Skin response: Died before resolution; Outcome: Died |
| 89 | 92 | [92] Gioe et al. (2024) | Case report | 62 / Male | Not reported | Site: Lung; Histology: Squamous cell carcinoma (SCC); TNM/Stage: Not reported; Metastasis: No | Before (10 months) | Distribution: Palms, soles; Nails: Trachyonychia (9 nails) | Spongiosis | Chemotherapy + radiotherapy | Skin response: Complete resolution; Outcome: Alive |
| 90 | 93 | [93] Mititelu & Powell (2019) | Case report | 72 / Female | Smoker | Site: Lung; Histology: Non-small cell lung cancer (NSCLC); TNM/Stage: Not reported; Metastasis: No | Before (4 months) | Distribution: Palms, soles; Nails: Thickened, yellow nails | Parakeratosis | Lobectomy | Skin response: Complete resolution; Outcome: Alive |
| 91 | 94 | [94] Witkowski & Parish (1982) | Case report | 65 / Male | Not reported | Site: Oropharynx; Histology: Squamous cell carcinoma (SCC); TNM/Stage: Advanced (metastatic); Metastasis: Yes | Before (2 months) | Distribution: Nose, cheeks; Nails: Grooved, shedding | Fibrinoid degeneration | Radiotherapy | Skin response: Cleared; Outcome: Died |
| 92 | 95 | [95] Lomholt et al. (2000) | Case series | 53 / Male | Smoker | Site: Lung; Histology: Adenocarcinoma; TNM/Stage: Advanced (metastatic); Metastasis: Yes | Before (2.5 years) | Distribution: Palms, trunk; Nails: Onycholysis | Psoriasiform changes | Pneumonectomy | Skin response: Complete resolution; Outcome: Alive |
| 93 | 95 | [95] Lomholt et al. (2000) | Case series | 60 / Female | Smoker | Site: Lung; Histology: Plano-cellular carcinoma; TNM/Stage: Not reported; Metastasis: No | Before (18 months) | Distribution: Generalized; Nails: Not reported | Chronic inflammation and dermatophytes | Resection | Skin response: Complete resolution; Outcome: Alive |
| 94 | 96 | [96] Strobel et al. (2006) | Case report | 58 / Male | Alcohol use | Site: Lung; Histology: Squamous cell carcinoma (SCC); TNM/Stage: Advanced (metastatic); Metastasis: Yes | Before | Distribution: Nose, ears; Nails: Hyperkeratosis | Spongiosis | Resection + radiotherapy | Skin response: Complete resolution; Outcome: Alive |
| 95 | 97 | [97] George et al. (2017) | Case report | 79 / Female | Smoker | Site: Lung; Histology: Squamous cell carcinoma (SCC); TNM/Stage: Not reported; Metastasis: No | Before (5 months) | Distribution: Hands, feet, legs; Nails: Brown discoloration, detachment | Spongiosis | Lobectomy | Skin response: Partial improvement; Outcome: Alive |
| 96 | 98 | [98] O'Keeffe et al. (2020) | Case report | 53 / Female | History of lung cancer | Site: Lung; Histology: Non-small cell lung cancer (NSCLC); TNM/Stage: Not reported; Metastasis: No | After (during pembrolizumab) | Distribution: Limbs; Nails: Onycholysis | Spongiosis | Corticosteroids | Skin response: Not reported; Outcome: Alive |
| 97 | 99 | [99] Hara et al. (1995) | Case report | 54 / Male | Scar | Site: Skin; Histology: Cutaneous squamous cell carcinoma; TNM/Stage: Not reported; Metastasis: No | Before (3 months) | Distribution: Nose, palms; Nails: Nail dystrophy | Elongation of rete ridges | Excision | Skin response: Complete resolution; Outcome: Alive |
| 98 | 100 | [100] Driessen et al. (2013) | Case report | 61 / Male | Hypopharyngeal cancer | Site: Hypopharynx; Histology: Squamous cell carcinoma (SCC); TNM/Stage: Stage IVA (pT4N2bM0); Metastasis: Yes | Before (6 months) | Distribution: Hands, nose, ears; Nails: Not reported | Blisters, interface dermatitis | Dexamethasone | Skin response: Partial improvement; Outcome: Died |
| 99 | 101 | [101] Van Leersum (2015) | Case report | 66 / Male | Weight loss | Site: Oropharynx; Histology: Squamous cell carcinoma (SCC); TNM/Stage: Stage IVA (T4aN2cM0); Metastasis: Yes | Before (4 weeks) | Distribution: Ears (painful), palms; Nails: Not reported | Not reported | Chemoradiotherapy | Skin response: Complete resolution; Outcome: Died |
| 100 | 102 | [102] Cicek et al. (2011) | Case report | 60 / Male | History of lung cancer | Site: Lung; Histology: Metastatic malignancy (unknown primary); TNM/Stage: Advanced (metastatic); Metastasis: Yes | Before (6 months) | Distribution: Flexural (axilla), pustules; Nails: Paronychia, subungual hyperkeratosis, yellow discoloration, oncholysis | Pustules, spongiosis | Corticosteroids | Skin response: Died before resolution; Outcome: Died |
| 101 | 103 | [103] Wishart (1986) | Case report | 64 / Male | Smoker | Site: Unknown primary; Histology: Squamous cell carcinoma (cervical); TNM/Stage: Advanced (metastatic); Metastasis: Yes | After (2 years) | Distribution: Hands, nose; Nails: Clubbing | Psoriasiform changes | Etretinate | Skin response: Complete resolution; Outcome: Died |
| 102 | 104 | [104] Sharma et al. (2006) | Case report | 63 / Female | Smoker | Site: Lung; Histology: Bronchogenic carcinoma; TNM/Stage: Advanced (metastatic); Metastasis: Yes | Before (10 months) | Distribution: Hands, feet, right ear; Nails: Not reported | Hyperkeratosis, acanthosis, parakeratosis and a perivascular lympho-histiocytic infiltrate in the upper dermis | Not reported | Skin response: Not reported; Outcome: Not reported |
| 103 | 105 | [105] Ikeda et al. (2022) | Case report | 77 / Male | Smoker | Site: Lung; Histology: Lung cancer; TNM/Stage: Not reported; Metastasis: No | Before (2 months) | Distribution: Palms, soles; Nails: Not reported | Not reported | Surgical resection | Skin response: Complete resolution; Outcome: Alive |
| 104 | 106 | [106] Dabas et al. (2018) | Case report | 50s / Male | Smoker | Site: Tongue; Histology: Squamous cell carcinoma (SCC); TNM/Stage: Stage IV (T2N2cM1); Metastasis: Yes | Before (5 weeks) | Distribution: Ears, hands; Nails: Subungual hyperkeratosis | Apoptotic keratinocytes | Palliative care | Skin response: Died before resolution; Outcome: Died |
| 105 | 107 | [107] Asokan et al. (1996) | Case report | 60 / Male | Smoker | Site: Hypopharynx; Histology: Squamous cell carcinoma (SCC); TNM/Stage: T4N2M1; Metastasis: Yes | Before (1 month) | Distribution: Palms, soles; Nails: Longitudinal bands, subungal hperkeratosis | Hyperkeratosis, parakeratosis, lymphocytic collection, histiocytes | Not reported | Skin response: Not reported; Outcome: Not reported |
| 106 | 108 | [108] Villares-Soriano (2014) | Case report | 67 / Male | Smoker | Site: Hypopharynx; Histology: Squamous cell carcinoma (SCC); TNM/Stage: Not reported; Metastasis: Not reported | Before (2 months) | Distribution: Hands, elbows; Nails: Not reported | Not reported | Resection | Skin response: Complete resolution; Outcome: Alive |
